# Two Small RNAs Conserved in *Enterobacteriaceae* Provide Intrinsic Resistance to Antibiotics Targeting the Cell Wall Biosynthesis Enzyme Glucosamine-6-Phosphate Synthase

**DOI:** 10.3389/fmicb.2016.00908

**Published:** 2016-06-15

**Authors:** Muna A. Khan, Yvonne Göpel, Slawomir Milewski, Boris Görke

**Affiliations:** ^1^Department of Microbiology, Immunobiology and Genetics, Max F. Perutz Laboratories, University of Vienna, Vienna BiocenterVienna, Austria; ^2^Department of Pharmaceutical Technology and Biochemistry, Faculty of Chemistry, Gdańsk University of TechnologyGdańsk, Poland

**Keywords:** glucosamine-6-phosphate synthase GlmS, Nva-FMDP, Bacilysin, intrinsic resistance, small RNA, GlmY, GlmZ, antimicrobial chemotherapy

## Abstract

Formation of glucosamine-6-phosphate (GlcN6P) by enzyme GlcN6P synthase (GlmS) represents the first step in bacterial cell envelope synthesis. In *Escherichia coli*, expression of *glmS* is controlled by small RNAs (sRNAs) GlmY and GlmZ. GlmZ activates the *glmS* mRNA by base-pairing. When not required, GlmZ is bound by adapter protein RapZ and recruited to cleavage by RNase E inactivating the sRNA. The homologous sRNA GlmY activates *glmS* indirectly. When present at high levels, GlmY sequesters RapZ by an RNA mimicry mechanism suppressing cleavage of GlmZ. The interplay of both sRNAs is believed to adjust GlmS synthesis to the needs of the cell, i.e., to achieve GlcN6P homeostasis. Bacilysin (tetaine) and Nva-FMDP are dipeptide antibiotics that impair cell envelope synthesis by inhibition of enzyme GlmS through covalent modification. However, although taken up efficiently, these antibiotics are less active against *E. coli* for reasons unknown so far. Here we show that the GlmY/GlmZ circuit provides resistance. Inhibition of GlmS causes GlcN6P deprivation leading to activation of GlmY and GlmZ, which in turn trigger *glmS* overexpression in a dosage-dependent manner. Mutation of *glmY* or *glmZ* disables this response and renders the bacteria highly susceptible to GlmS inhibitors. Thus, *E. coli* compensates inhibition of GlmS by increasing its synthesis through the GlmY/GlmZ pathway. This mechanism is also operative in *Salmonella* indicating that it is conserved in *Enterobacteriaceae* possessing these sRNAs. As GlmY apparently responds to GlcN6P, co-application of a non-metabolizable GlcN6P analog may prevent activation of the sRNAs and thereby increase the bactericidal activity of GlmS inhibitors against wild-type bacteria. Initial experiments using glucosamine-6-sulfate support this possibility. Thus, GlcN6P analogs might be considered for co-application with GlmS inhibitors in combined therapy to treat infections caused by pathogenic *Enterobacteriaceae*.

## Introduction

**FIGURE 1:**
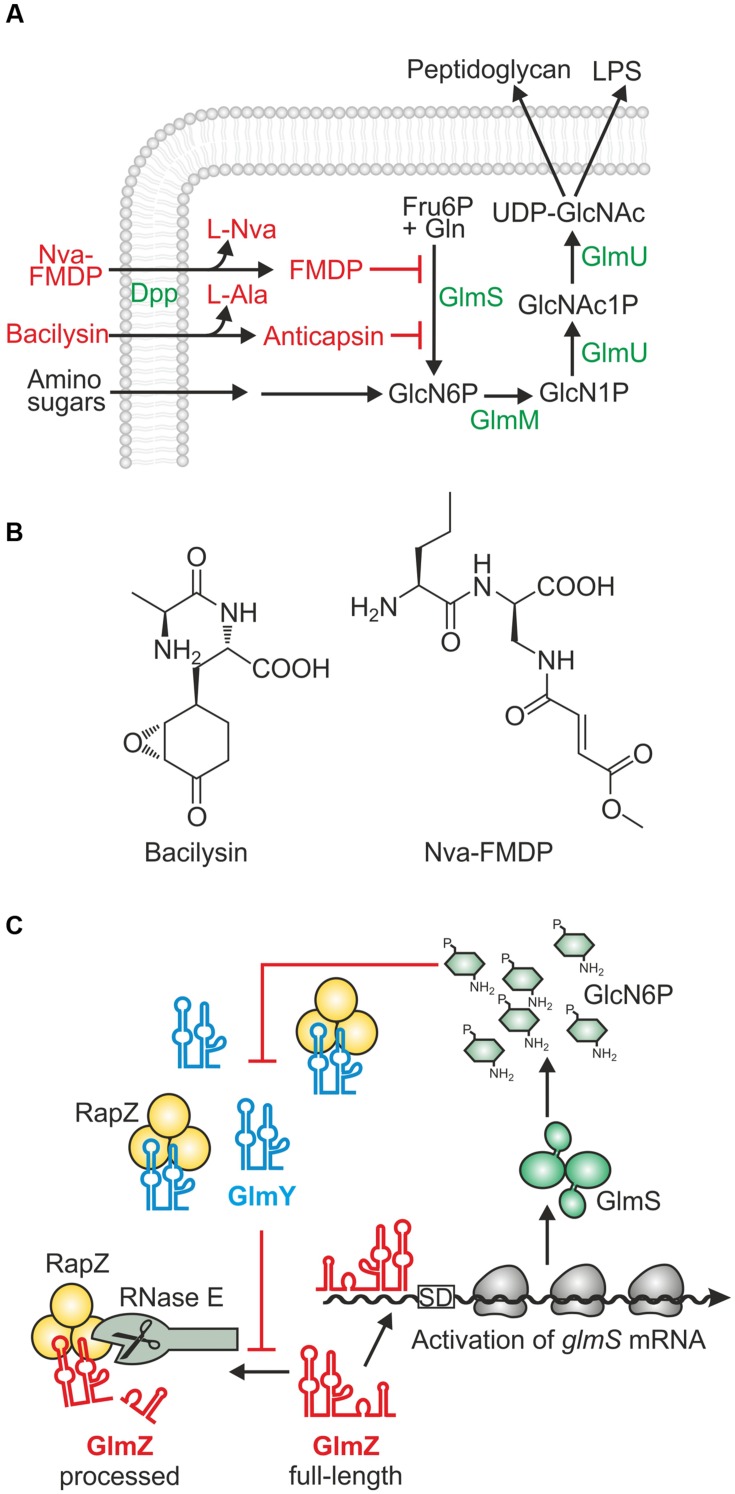
**Role, regulation and inhibitors of enzyme GlmS in *Escherichia coli*. (A)** Key role of enzyme GlmS in the hexosamine pathway and its inhibition by antibiotics. GlmS catalyzes synthesis of GlcN6P from fructose-6-phosphate and glutamine. When available, amino sugars such as GlcN can be taken up and converted to GlcN6P bypassing the need for GlmS. GlcN6P is further converted by enzymes GlmM and GlmU to UDP-GlcNAc, which is the last common precursor for synthesis of peptidoglycan and lipopolysaccharides (LPS). The dipeptide antibiotics Nva-FMDP and bacilysin require peptide transporters such as Dpp for uptake. Intracellular hydrolysis releases the active C-terminal moieties FMDP and anticapsin, which inhibit GlmS through covalent binding to a residue in the glutamine binding pocket. **(B)** Structures of the dipeptide antibiotics bacilysin and Nva-FMDP. **(C)** Feedback regulation of GlmS synthesis by small RNAs GlmY and GlmZ and adapter protein RapZ in *E. coli* (Göpel et al., 2013, 2016). GlmZ base-pairs with the *glmS* 5′-UTR enhancing translation and stabilizing the transcript. Alternatively, GlmZ is bound by adapter protein RapZ and recruited to cleavage by RNase E. The decision on the fate of GlmZ is made by the homologous decoy sRNA GlmY. Upon GlcN6P scarcity, GlmY accumulates and sequesters RapZ thereby counteracting cleavage of GlmZ by RNase E.

The continuous increase and spread of antibiotic resistance mechanisms among bacterial pathogens represents a major threat for public health. Of particular concern is the recent emergence of extended spectrum β-lactamases and carbapenemases in pathogenic *Enterobacteriaceae* limiting therapeutic treatment options for infections caused by these bacteria. Thus, there is an urgent need for novel therapies, which may not only include the discovery of novel antibacterial drugs, but also revision of known compounds that were previously neglected (Brown and Wright, 2016; Mühlen and Dersch, 2016). Many clinically relevant antibiotics interfere with the biochemical machinery for peptidoglycan biosynthesis (Silver, 2013; Borisova et al., 2014). However, the initial steps in this pathway collectively referred to as hexosamine pathway, have been rarely considered as drug targets. The hexosamine pathway generates UDP–*N*-acetyl-glucosamine (UDP–GlcNAc) and is initiated by key enzyme glucosamine-6-phosphate (GlcN6P) synthase GlmS, which synthesizes GlcN6P from L-glutamine and fructose-6-phosphate (**Figure 1A**; Milewski, 2002; Teplyakov et al., 2002). GlmS is essential unless exogenous amino sugars such as glucosamine (GlcN) are available in adequate amounts. GlcN can be taken up and converted to GlcN6P, thereby bypassing GlmS (**Figure 1A**) (Plumbridge, 2015). The GlcN concentration in human bodily fluids is too low to sustain growth of *glmS* mutants making GlmS essential for enteric bacteria colonizing the human host (Persiani et al., 2007; Kim et al., 2013; Bennett et al., 2016).

Several naturally produced antibiotics that inhibit GlmS enzymatic activity have been identified including bacilysin and compound A 19009 synthesized by *Bacillus subtilis* and *Streptomyces collinus*, respectively (Molloy et al., 1972; Kenig and Abraham, 1976). These antibiotics are dipeptides of exotic amino acids and rely on peptide transporters for uptake and on intracellular peptidases for release of the warhead moiety, which is anticapsin in case of bacilysin (**Figures 1A,B**; Milewski, 2002; Özcengiz and Ögülür, 2015). A systematic survey of synthetic derivatives related to A 19009 identified the corresponding methylester *N*^3^-(4-methoxyfumaroyl)-(*S*)-2,3-diaminopropanionic acid (FMDP) as most efficient and selective inhibitor of purified GlmS from various organisms including *Escherichia coli* and *Salmonella* (Chmara et al., 1986; Badet et al., 1988). Among various tested FMDP peptides, L-norvalyl-FMDP (Nva-FMDP; **Figures 1A,B**) exhibited the strongest growth inhibitory effect on bacteria (Andruszkiewicz et al., 1987; Chmara et al., 1998). FMDP as well as anticapsin act as glutamine analogs and covalently bind to the glutamine binding domain of GlmS causing its irreversible inhibition (Milewski et al., 1986; Kucharczyk et al., 1990). As a result, GlcN6P production is blocked leading to exhaustion of nucleotide precursors for peptidoglycan biosynthesis and ultimately to bacteriolysis. Cell death can be prevented by co-administration of amino sugars demonstrating that these antibiotics are specific for GlmS and lack off-target activity (Kenig and Abraham, 1976; Chmara et al., 1998). Nva-FMDP is highly effective against Gram-positive bacteria, but shows only weak activity against *E. coli* [minimal inhibitory concentration (MIC) ≥ 100 μg/ml; Andruszkiewicz et al., 1987; Chmara et al., 1998], although it is taken up rapidly and efficiently by the Dpp dipeptide ATP binding cassette (ABC) transporter (Marshall et al., 2003). So far, the reason for this weak efficacy remained mysterious.

Synthesis of GlmS is feed-back regulated by GlcN6P, thereby achieving homeostasis of this metabolite. The underlying mechanisms employ regulatory RNA elements, but differ remarkably between Gram-positive and Gram-negative bacteria. The *glmS* mRNA of Gram-positive species contains a ribozyme in its 5′-untranslated region (5′-UTR), which upon binding of GlcN6P triggers self-cleavage leading to down-regulation of *glmS* expression (Winkler et al., 2004). In contrast, *E. coli* and presumably most species of the Gram-negative *Enterobacteriaceae* employ two trans-encoded homologous small RNAs (sRNAs), GlmY and GlmZ, and adapter protein RapZ to regulate GlmS synthesis (**Figure 1C**) (Reichenbach et al., 2008; Urban and Vogel, 2008; Göpel et al., 2013, 2016). Assisted by RNA chaperone Hfq, GlmZ base-pairs with the 5′-UTR of the *glmS* transcript and stimulates translation concomitantly stabilizing the mRNA. In an alternative fate, GlmZ is bound by protein RapZ, which recruits RNase E to inactivate the sRNA through processing. The path to be taken by GlmZ is ultimately determined by the level of sRNA GlmY. GlmY accumulates when GlcN6P decreases in the cell and sequesters RapZ through molecular mimicry. As a result, GlmZ remains un-cleaved and upregulates *glmS* expression to replenish GlcN6P. In addition, in enterohemorrhagic *E. coli* GlmY and GlmZ were recruited to regulate horizontally acquired virulence genes (Gruber and Sperandio, 2014, 2015).

In the present study, we investigated the roles of GlmY and GlmZ for susceptibility to GlmS inhibitors. We show that these sRNAs provide intrinsic resistance by countervailing inhibition of GlmS with its increased synthesis. Beyond *E. coli*, this mechanism also operates in *Salmonella* indicating that it applies to a wider range of enterobacterial species in which these sRNAs are conserved. Our findings indicate that compounds suppressing GlmY and/or GlmZ would increase the efficacy of GlmS inhibitors when applied in combination. Experiments using the non-metabolizable GlcN6P analog glucosamine-6-sulfate (GlcN6SO_4_) are in favor of this possibility.

## Materials and Methods

### Bacterial Strains, Culture Conditions, and Chemicals

Bacterial strains used in this study are listed in **Table 1**. Derivatives of *E. coli* MG1655 were used to test the bacteriocidal effects of Nva-FMDP and bacilysin. In these strains the *lacZ* gene is deleted allowing monitoring of *lacZ* reporter gene fusions. General transduction by phage T4GT7 (Wilson et al., 1979) was used to introduce established *ΔglmY::cat*, *ΔglmZ::cat* alleles and the spectinomycin resistance tagged *glmS’–lacZ* fusion into strain S4197, respectively. The temperature-sensitive Flippase (FLP)-recombinase plasmid pCP20 was used to remove the chloramphenicol resistance genes (Datsenko and Wanner, 2000). Additional experiments were performed using *Salmonella enterica* serovar *Typhimurium* strain SL1344 and its *ΔglmY* and *ΔglmZ* derivatives JVS112 and JVS122, respectively (Papenfort et al., 2008). Bacteria were grown at 37°C in Luria-Bertani (LB) Lennox, Mueller Hinton broth (Merck), MacConkey lactose (Roth), and M9 minimal medium (Miller, 1972). M9 medium was supplemented with 1% [w/v] glucose as carbon source and in case of *Salmonella* strains additionally with 0.002% L-histidine. Antibiotics were added when required: chloramphenicol (15 μg/ml), spectinomycin (75 μg/ml), neomycin (4 μg/ml), erythromycin (0.5 μg/ml). Nva-FMDP was synthesized as described previously (Andruszkiewicz et al., 1987) and solved in H_2_O to yield a stock solution of 1 mg/ml. D-GlcN6SO_4_ was purchased from Santa Cruz Biotechnology. *B. subtilis* strain TI304, which overproduces bacilysin, was used to prepare bacilysin enriched culture supernatant as previously described (Inaoka et al., 2009). Bacilysin-free supernatant, which served as negative control, was prepared using *B. subtilis* strain TI398. The latter strain is unable to synthesize bacilysin due to inactivation of gene *ywfE* (*bacD*) encoding alanine–anticapsin ligase (Inaoka et al., 2009). The culture supernatants were concentrated 20-fold using a centrifugal evaporator and subsequently stored at -20°C until further use.

**Table 1:** Bacterial strains used in this study.

Name	Genotype	Reference/construction
***Bacillus subtilis***		
TI304	168 *ΔscoC::spc*, *abrB::neo*, *codY::erm*	(Inaoka et al., 2009)
TI398	168 *ywfA::neo*, *ywfE179*	(Inaoka et al., 2009)
***Escherichia coli***		
S4197	MG1655 *rph^+^, ilvG^+^, ΔlacZ*	(Venkatesh et al., 2010)
Z8	CSH50 *Δ(pho-bgl)201*, *Δ(lac-pro)*, *ara*, *thi*, *λttB*::[*aadA* (spec^R^), *glmS* -5′::*lacZ*], *strp^R^, F’*(*lacI^q^*)	(Kalamorz et al., 2007)
Z44	CSH50 *Δ(pho-bgl)201*, *Δ(lac-pro)*, *ara*, *thi*, *ΔglmZ::cat*	(Kalamorz et al., 2007)
Z95	CSH50 *Δ(pho-bgl)201*, *Δ(lac-pro)*, *ara*, *thi*, *ΔglmY::cat*	(Reichenbach et al., 2008)
Z834	S4197 *ΔglmY::cat*	T4GT7 (Z95) → S4197; this work
Z835	S4197 *ΔglmZ::cat*	T4GT7 (Z44) → S4197; this work
Z838	S4197 *ΔglmY*	Z834 cured from *cat*; this work
Z839	S4197 *ΔglmZ*	Z835 cured from *cat*; this work
Z854	S4197 *λttB*::[*aadA* (spec^R^), *glmS* -5′::*lacZ*]	T4GT7 (Z8) → S4197; this work
Z855	S4197 *λttB*::[*aadA* (spec^R^), *glmS* -5′::*lacZ*], *ΔglmY*	T4GT7 (Z8) → Z838; this work
Z856	S4197 *λttB*::[*aadA* (spec^R^), *glmS* -5′::*lacZ*], *ΔglmZ*	T4GT7 (Z8) → Z839; this work
***Salmonella enterica* serovar *Typhimurium***		
SL1344	Str^R^, *his*, *rpsL*, *xyl*	(Hoiseth and Stocker, 1981)
JVS112	SL1344 *ΔglmY*	(Papenfort et al., 2008)
JVS122	SL1344 *ΔglmZ*	(Papenfort et al., 2008)

### Large Scale Liquid Culture Experiments to Study the Effects of Nva-FMDP and Bacilysin

Large scale cultures were grown to assess the effect of Nva-FMDP and Bacilysin on cell viability and the regulatory GlmY/GlmZ/*glmS* circuit. To test Nva-FMDP, strains were inoculated from overnight cultures in 35 ml LB medium to an OD_600_ ~ 0.1 and grown until an OD_600_ ~ 0.3. Subsequently, cultures were split into three 10 ml subcultures that were supplemented either with 100 μg/ml Nva-FMDP, 100 μg/ml Nva-FMDP + 0.2% GlcN or with the equivalent volume of H_2_O (mock control), respectively. Growth was continued for 6 h and 1.5 ml samples were taken in hourly intervals for RNA extraction and to determine the OD_600_, CFU numbers and β-galactosidase activities. A similar setup was used to test bacilysin-containing culture supernatant but as a difference cultures were split at an OD_600_ ~ 0.2 and supplemented either with 1.5 ml concentrated culture supernatant of *B. subtilis* strains TI304 or TI398 or with H_2_O (mock control), respectively.

### Determination of CFU Numbers

To determine CFU numbers, serial dilutions of the cultures were prepared in Mg saline (145 mM NaCl, 10 mM MgSO_4_) and appropriate dilutions were plated in duplicate on non-selective LB agar. Following incubation at 37°C overnight, colonies were counted from plates containing 30–300 colonies using a Stuart SC6 colony counter (Bibby Scientific).

### Extraction of Total RNA and Northern Blotting

Total RNA was extracted from 1 ml samples using the RNeasy mini kit (Qiagen) according to the instructions of the manufacturer. The RNAs of interest were detected in subsequent Northern experiments using DIG labeled RNA probes that were generated by *in vitro* transcription using the DIG labeling kit (Roche diagnostics). The corresponding DNA templates were obtained by PCR using previously described oligonucleotide primers (Reichenbach et al., 2008, 2009). To detect sRNAs GlmY and GlmZ, 2.5 μg total RNA per lane were separated on 8% polyacrylamide gels containing 7 M urea and 1 × TBE. Subsequently, RNA was transferred to a nylon membrane (Hybond N^+^, GE healthcare) by electro-blotting and cross-linked by 254 nm UV radiation. RNA hybridization and detection was performed according the supplier’s instruction (Roche Diagnostics). To obtain 5S loading controls, sRNA probes were stripped by incubating the membrane 2× for 60 min in 50% formamide, 5% SDS, 50 mM Tris-HCl pH 7.5. Subsequently, the membrane was re-probed using a probe directed against the *rrfD* transcript. For detection of *glmS* mRNA, 5 μg total RNA per lane were separated by formaldehyde agarose gel electrophoresis (1% agarose, 18% formaldehyde, 20 mM MOPS, 5 mM sodium acetate, 1 mM EDTA, pH 7.0) and subsequently blotted onto a Hybond N^+^ nylon membrane using the VacuGene XL vacuum blotting system (Amersham Biosciences) at 80 mbar for 4 h. To obtain loading controls, the 23S and 16S rRNAs were visualized by ethidium bromide prior to blotting.

### β-Galactosidase Assays

β-Galactosidase enzyme activities were determined as described previously (Miller, 1972). Enzyme activities are expressed in Miller units and represent the average of at least two measurements using independent cultures.

### Disk Diffusion Assay

Disk diffusion assays (Bauer et al., 1966) were carried out on various media including Mueller Hinton, LB Lennox, MacConkey lactose and M9 agar. Cells were grown at 37°C in LB to an OD_600_ ~ 0.5, pelleted and re-suspended in the medium subsequently used in the assay. A volume containing ~4 × 10^7^ cells was mixed with 4 ml thin agar layer (medium containing 7.5% agarose) that was kept molten at 44°C and subsequently spread over 15 ml solid agar base of the same medium prepared in 100 mm Petri dishes. The compounds to be tested were adsorbed onto 6 mm cellulose disks (Rotilabo), which were subsequently placed on the seeded agar surface in the Petri dishes. Growth inhibition zones were recorded following incubation for 16 h at 37°C. Assays were performed at least twice using independent cultures.

### Determination of MICs

Minimal inhibitory concentrations were determined in various media according the guidelines of the European society of clinical microbiology and infectious diseases using the broth microdilution method (EUCAST, 2003). Briefly, serial dilutions of the compounds to be tested were prepared in 96 well plates (Sterilin) and subsequently inoculated with 5 × 10^5^ CFU/ml of an overnight culture grown in the same medium as used for MIC determination. Following incubation at 37°C for 12 h growth was monitored spectrophotometrically by measuring the OD_595_ using a microplate reader (iMark plate reader, BioRad). MIC was defined by the lowest drug concentration preventing visible growth (OD_595_ < 0.1). MIC assays were performed at least twice using independent cultures.

## Results

### *Escherichia coli* Responds to Nva-FMDP by Overexpression of *glmS*

**FIGURE 2:**
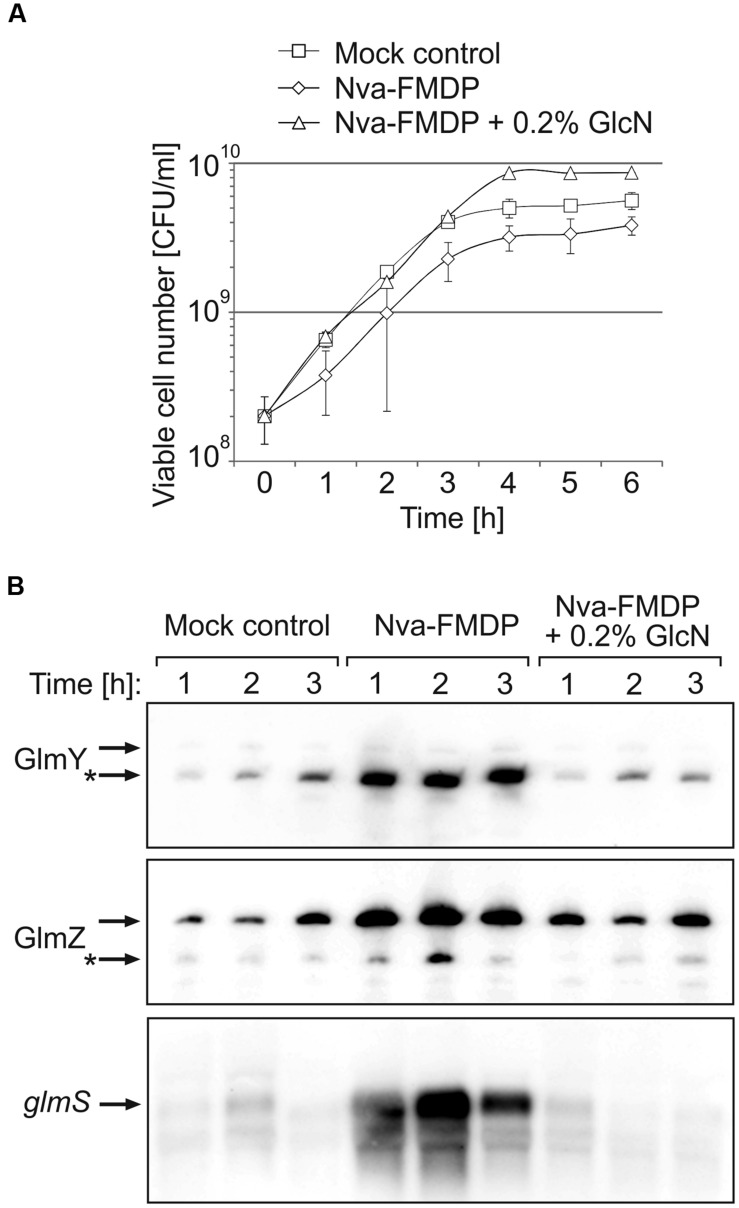
**Nva-FMDP triggers accumulation of GlmY, GlmZ and *glmS* RNAs in *E. coli* and co-administration of GlcN suppresses this effect. (A)** CFU numbers of cultures of *E. coli* strain Z854 grown in LB supplemented either with 100 μg/ml Nva-FMDP (diamonds) or 100 μg/ml Nva-FMDP + 0.2% glucosamine (triangles) or the equivalent volume of H_2_O (mock control; squares). The corresponding OD_600_ readings are provided in Supplementary Figure S1A. **(B)** Northern analysis of GlmY, GlmZ, and *glmS* RNAs in the cultures analyzed in **(A)**. Total RNA was extracted from samples harvested at hourly intervals during the first 3 h after inoculation and subsequently probed for GlmY (top panel), GlmZ (medium panel) and *glmS* RNAs (bottom panel). Loading controls are provided in Supplementary Figure S1B. The various RNA species are indicated by arrows. Processed variants are denoted by asterisks.

Nva-FMDP exerts only a moderate growth inhibitory effect on *E. coli* cells (Andruszkiewicz et al., 1987). Our preliminary results suggested that *E. coli* responds to this drug by upregulation of GlmS synthesis (Reichenbach et al., 2008). To gain further insight, we first studied the effect of Nva-FMDP at sub-MIC concentrations. To this end, we used strain Z854, a derivative of *E. coli* K-12 reference strain MG1655 carrying an ectopic *glmS’–lacZ* fusion on the chromosome. Strain Z854 was grown in LB Lennox supplemented with either 100 μg/ml Nva-FMDP, or 100 μg/ml Nva-FMDP + 0.2% GlcN or with the corresponding volume of water (mock control). OD_600_ readings detected no growth impairment by Nva-FMDP (Supplementary Figure S1A). However, the CFU number was diminished ~twofold as compared to the mock control (**Figure 2A**). Addition of GlcN cured this growth defect, corroborating that Nva-FMDP causes cell death by depletion of intracellular GlcN6P resulting from GlmS inhibition. To determine *glmS* expression levels, total RNA was extracted from cells harvested during the first 3 h of growth and analyzed by Northern blotting. In parallel, the β-galactosidase activities were determined. Both assays consistently detected a strong upregulation of *glmS* expression in the Nva-FMDP treated cells, which was suppressed by the addition of GlcN (**Figures 2B**, bottom panel and **4B**, columns left). To determine whether GlmY and GlmZ are involved in upregulation of *glmS* expression in response to Nva-FMDP, we performed Northern blotting. Indeed, high levels of GlmY and of full-length GlmZ were observed in the Nva-FMDP treated cells but not in the other samples (**Figure 2B**).

### Activation of the GlmY/GlmZ/*glmS* Regulatory Cascade Is Not Restricted to Nva-FMDP But Applies to GlmS Inhibitors in General

**FIGURE 3:**
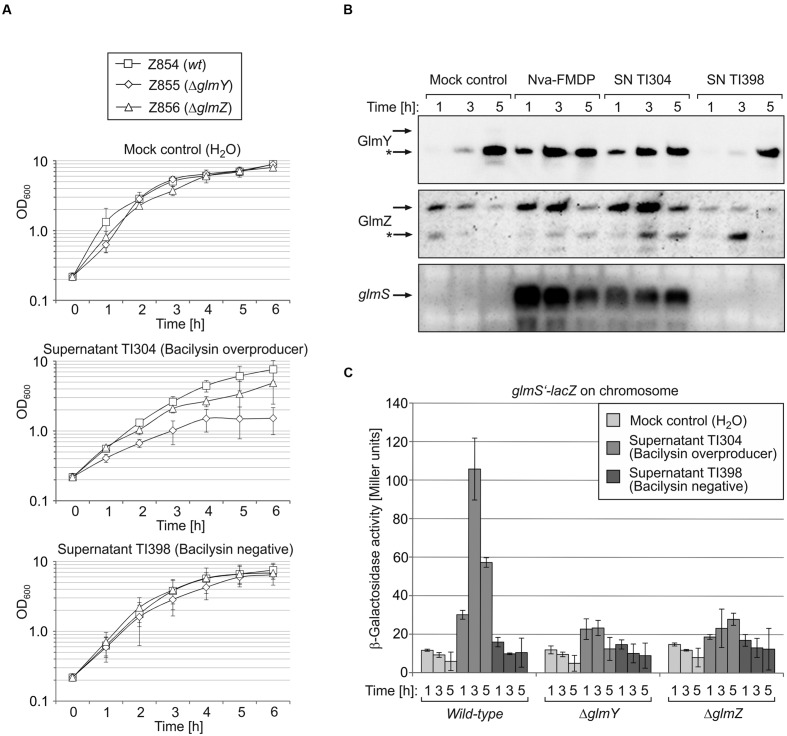
**Bacilysin causes accumulation of GlmY, GlmZ and *glmS* RNAs in *E. coli* recapitulating the effects of Nva-FMDP.** *E. coli* strains Z854 (wild-type), Z855 (*ΔglmY*) and Z856 (*ΔglmZ*) were grown in LB supplemented either with culture supernatant (SN) of *B. subtilis* strain TI304 overproducing bacilysin or with SN derived from the bacilysin-negative *B. subtilis* mutant TI398. Cultures treated with the equivalent volume of H_2_O served as mock control. **(A)** Growth curves of the various cultures as determined by OD_600_ recording. **(B)** Northern blot analysis for detection of GlmY, GlmZ and *glmS* in the cultures of *E. coli* wild-type strain Z854. Samples for isolation of total RNA were harvested 1, 3, and 5 h after inoculation of the cultures. For comparison, a culture of strain Z854 treated with Nva-FMDP was analyzed in parallel. Loading controls are provided in Supplementary Figure S2. It should be noted that expression of *glmY* is generally upregulated in *E. coli* when entering the stationary phase (Göpel et al., 2013) explaining accumulation of GlmY in the mock control culture following 5 h of growth. However, the high GlmY levels are not transduced to *glmS* due to the low expression levels of GlmZ under these conditions. **(C)** β-Galactosidase assays monitoring expression of a *glmS’–lacZ* reporter fusion in the various cultures. Enzyme activities were determined from samples harvested after 1, 3, and 5 h of growth.

Next, we determined whether upregulation of the GlmY/GlmZ/*glmS* regulatory cascade is specific to Nva-FMDP or may also apply to other GlmS inhibitors such as bacilysin. Since bacilysin is commercially unavailable, we used concentrated culture supernatant of *B. subtilis* strain TI304, which overproduces and secretes bacilysin into the medium (Inaoka et al., 2009). Culture supernatant of the bacilysin-negative *B. subtilis* mutant TI398 served as negative control. *E. coli* strain Z854 was grown in LB Lennox supplemented with supernatant of *B. subtilis* strain TI304 or TI398 or with water (mock control). Presence of the bacilysin containing supernatant resulted in slight growth retardation, which was not observable in the culture treated with supernatant of the bacilysin-negative mutant (**Figure 3A**). This observation indicated that the growth defect was specifically caused by bacilysin present in the culture supernatant of TI304. Northern blot analysis of RNA extracted from samples harvested at various times detected accumulation of the *glmS* transcript and of the GlmY/GlmZ sRNAs in cells treated with supernatant of TI304, similar to cells treated with Nva-FMDP, which was tested in parallel (**Figure 3B**). Upregulation of *glmS* in response to the bacilysin-containing culture supernatant was also confirmed by β-galactosidase assays (**Figure 3C**, columns left). In contrast, the culture supernatant of strain TI398 had no effect on GlmY, GlmZ, and *glmS* as judged from comparison with the mock control (**Figures 3B,C**). Taken together, the data indicate that activation of the GlmY/GlmZ system and overexpression of *glmS* is not a specific response to Nva-FMDP but applies to GlmS inhibitors in general.

### *E. coli* Mutants Lacking GlmY or GlmZ Fail to Upregulate *glmS* in Response to GlmS Inhibitors

**FIGURE 4:**
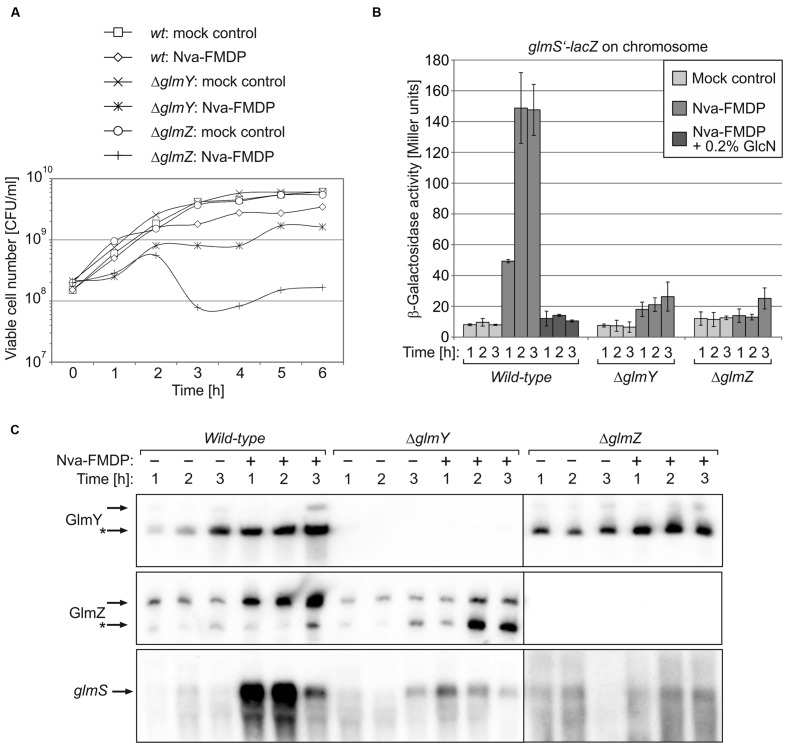
***Escherichia coli* mutants lacking GlmY or GlmZ do not respond to Nva-FMDP by upregulation of *glmS* mRNA levels.** *E. coli* strains Z854 (wild-type), Z855 (*ΔglmY*) and Z856 (*ΔglmZ*) were grown in LB supplemented either with Nva-FMDP or the equivalent volume of water (mock control), respectively. **(A)** CFU numbers of the various cultures. **(B)** β-Galactosidase assays monitoring expression of a *glmS’–lacZ* reporter fusion in the various cultures during the first 3 h following inoculation. **(C)** Northern analysis for detection of GlmY, GlmZ and *glmS* RNAs in the various cultures. For details, see legend of **Figure 2B**. Loading controls are provided in Supplementary Figure S3.

Northern analyses showed that Nva-FMDP and bacilysin trigger accumulation of sRNAs GlmY and GlmZ concomitantly with the *glmS* mRNA and additional presence of GlcN suppressed this effect (**Figures 2B** and **3B**). This observation suggested that intracellular depletion of GlcN6P caused by GlmS inhibition led to activation of the GlmY/GlmZ sRNAs, which in turn stimulated *glmS* expression. To prove this hypothesis, we studied the effect of Nva-FMDP on isogenic *E. coli ΔglmY* and *ΔglmZ* mutants, respectively. The strains were grown in the absence or presence of 100 μg/ml Nva-FMDP and samples were harvested at hourly intervals and analyzed. Northern blot analysis as well as determination of β-galactosidase activities revealed that both mutants failed to activate *glmS* expression in response to Nva-FMDP, whereas *glmS* strongly accumulated in the wild-type strain as observed before (**Figures 4B,C**). In the *ΔglmY* mutant treated with Nva-FMDP, stabilization of the full-length variant of GlmZ did not occur, explaining the inability to activate *glmS* (**Figure 4C**, middle). Vice versa, GlmY still accumulated in the *ΔglmZ* mutant upon exposure to Nva-FMDP, but remained without effect on *glmS*, which is explained by the absence of GlmZ (**Figure 4C**, right). As judged from *glmS’–lacZ* fusion data, the *ΔglmY* and *ΔglmZ* mutants also failed to activate *glmS* in response to bacilysin (**Figure 3C**). Therefore, these data show that antibiotics inhibiting GlmS induce *glmS* overexpression through activation of the GlmY/GlmZ regulatory sRNA cascade. Interestingly, growth monitoring suggested that both sRNA mutants are more susceptible to growth inhibition by Nva-FMDP and bacilysin as compared to the wild-type (**Figures 3A** and **4A**).

### Mutation of GlmY or GlmZ Attenuates Intrinsic Resistance of *E. coli* to Nva-FMDP

**FIGURE 5:**
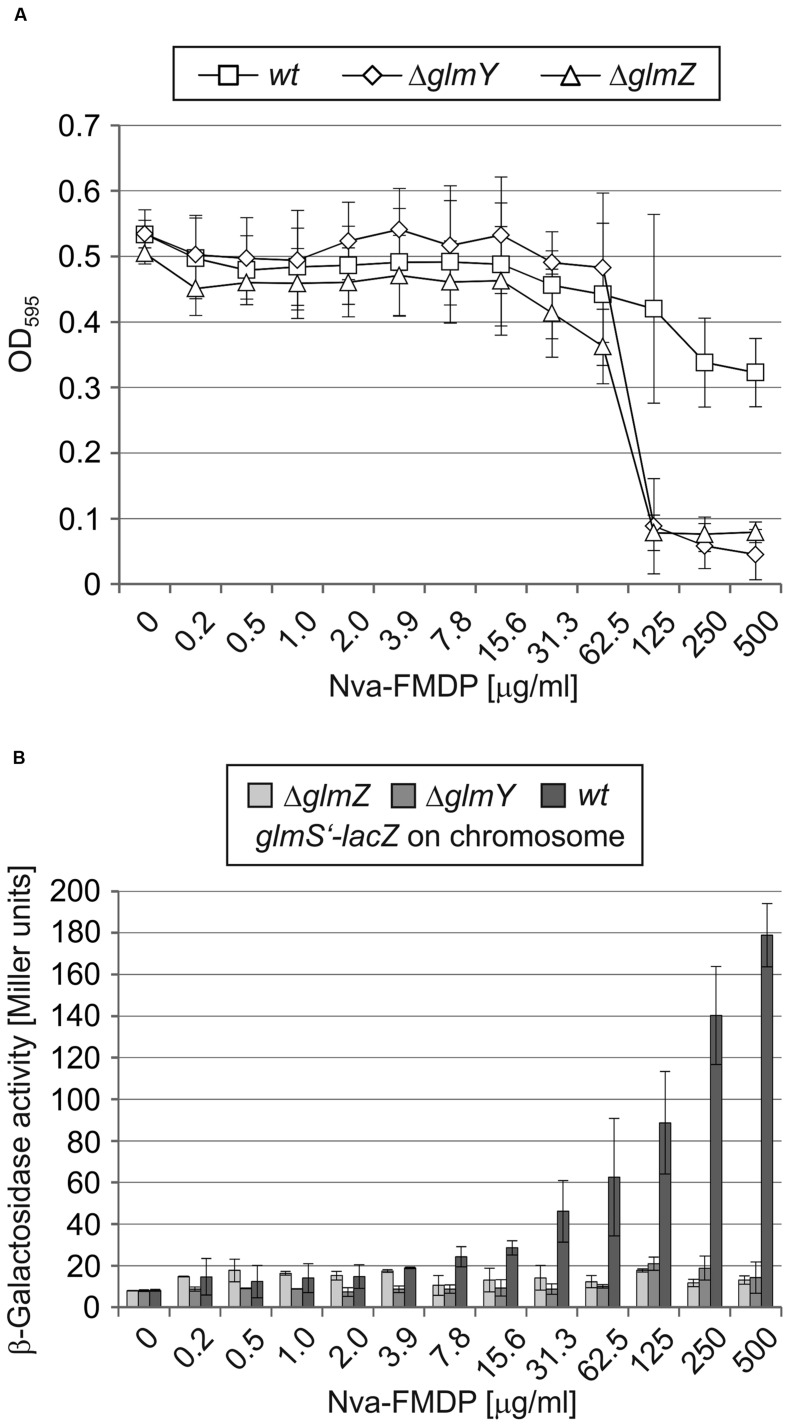
**GlmY and GlmZ enable *E. coli* to resist the bactericidal effect of Nva-FMDP by upregulation of *glmS* expression in a dosage-dependent manner.** Serial dilutions of Nva-FMDP in LB medium were inoculated with 5 × 10^5^ CFU/ml of *E. coli* strains Z854 (wild-type), Z855 (*ΔglmY*) and Z856 (*ΔglmZ*), respectively. **(A)** Cell densities of the cultures as determined by OD_595_ measurements after 12 h incubation. **(B)** β-Galactosidase activities monitoring expression of a chromosomal *glmS’–lacZ* fusion in the various cultures.

To investigate the possibly higher sensitivity of *ΔglmY* and *ΔglmZ* mutants to GlmS inhibitors, we first studied the effect of Nva-FMDP more thoroughly using the broth microdilution method. Serial dilutions of Nva-FMDP in LB medium were inoculated with the wild-type strain and the *ΔglmY* and *ΔglmZ* mutants, respectively. Following 12 h incubation, the OD_595_ of the cultures and the β-galactosidase activities were determined. Growth of the wild-type strain was slightly impaired only at highest Nva-FMDP concentrations (**Figure 5A**; MIC > 500 μg/ml). In contrast, growth of the mutant strains was completely inhibited at Nva-FMDP concentrations above 62.5 μg/ml. This higher susceptibility of the mutants can be attributed to the incapability to increase *glmS* expression as indicated by constantly low *glmS’–lacZ* expression levels regardless of the applied Nva-FMDP concentration (**Figure 5B**). In contrast, the wild-type strain exhibited *glmS’–lacZ* expression levels that gradually increased with incremental Nva-FMDP concentrations (**Figure 5B**). Thus, GlmY and GlmZ enable *E. coli* to activate *glmS* expression in a dosage-dependent manner, thereby overcoming inhibition of GlmS enzymatic activity by Nva-FMDP.

Next, we characterized susceptibility of the various *E. coli* strains to Nva-FMDP more systematically. To this end, we performed disk diffusion assays and MIC determinations in various media (**Tables 2** and **3**; Supplementary Figures S4A,B). Nva-FMDP showed no significant activity against the wild-type strain when grown in LB or Mueller Hinton medium. However, reasonable activity was detectable when the bacteria grew in MacConkey or M9 minimal medium, indicating that the antibacterial activity of Nva-FMDP is modulated by composition of the growth medium. In contrast to the wild-type, the *ΔglmY* and *ΔglmZ* mutants were growth-inhibited by Nva-FMDP in all media. Larger inhibition zones as compared to the wild-type were observed on the various disk diffusion plates and MIC values of the mutants were roughly fourfold lower demonstrating that absence of GlmY or GlmZ increases susceptibility to Nva-FMDP significantly (**Tables 2** and **3**, Supplementary Figure S4A,B). Again, susceptibility of the mutants to Nva-FMDP varied with the growth medium increasing in the order Mueller Hinton < LB < MacConkey lactose < M9 minimal medium. Co-administration of GlcN abolished growth inhibition by Nva-FMDP on the disk diffusion plates, regardless whether GlcN was included in the growth medium or directly administered on the filter disk (Supplementary Figures S4B,D), re-emphasizing that Nva-FMDP acts via depletion of metabolites of the amino sugar pathway.

**Table 2:** Disk diffusion assays addressing susceptibility of *Escherichia coli* and *Salmonella* strains to Nva-FMDP on various media.

	*Escherichia coli* K12			*Salmonella enterica* serovar *Typhimurium*		
Potency Nva-FMDP [μg]	Wild-type (Z854)	*ΔglmY* (Z855)	*ΔglmZ* (Z856)	Wild-type (SL1344)	*ΔglmY* (JVS112)	*ΔglmZ* (JVS122)
**LB Lennox**						
100	-	24 ± 1	24 ± 1	10 ± 1	18 ± 1	22 ± 0
20	-	20 ± 1	19 ± 4	-	12 ± 0	15 ± 0
10	-	14 ± 1	13 ± 1	-	9 ± 0	9 ± 0
**Mueller Hinton**						
100	-	10 ± 1	12 ± 1	10 ± 0	15 ± 0	16 ± 0
20	-	-	9 ± 1	-	12 ± 1	13 ± 0
10	-	-	-	-	9 ± 1	11 ± 0
**MacConkey, lactose**						
100	13 ± 1	27 ± 1	27 ± 1	21 ± 1	27 ± 0	30 ± 0
20	-	23 ± 1	22 ± 1	17 ± 1	23 ± 1	26 ± 1
10	-	19 ± 1	19 ± 2	13 ± 0	21 ± 1	23 ± 0
**M9 glucose**						
5	13 ± 1	19 ± 1	22 ± 2	14 ± 4	19 ± 1	24 ± 0
2	9 ± 1	15 ± 1	20 ± 3	13 ± 0	16 ± 2	22 ± 0
1	-	13 ± 1	19 ± 4	11 ± 1	13 ± 2	20 ± 0

*Diameters of zones of growth inhibition (mm) are recorded.*

**Table 3:** Susceptibility [minimal inhibitory concentration (MIC) in μg/ml] of *E. coli* and *Salmonella* strains to Nva-FMDP in various growth media.

	*Escherichia coli* K12			*Salmonella enterica* serovar *Typhimurium*		
Medium	Wild-type (Z854)	*ΔglmY* (Z855)	*ΔglmZ* (Z856)	Wild-type (SL1344)	*ΔglmY* (JVS112)	*ΔglmZ* (JVS122)
LB Lennox	>500	125	125	500	31.3	7.8
Mueller Hinton	>500	>500	>500	125	25	6.25
MacConkey, lac	6.25	1.56	0.78	3.13	0.39	0.2
M9 glucose	0.16	0.04	0.08	0.31	0.08	0.01

### The Mechanism of Intrinsic Resistance to GlmS Inhibitors Procured by GlmY and GlmZ Is Also Operative in *Salmonella*

**FIGURE 6:**
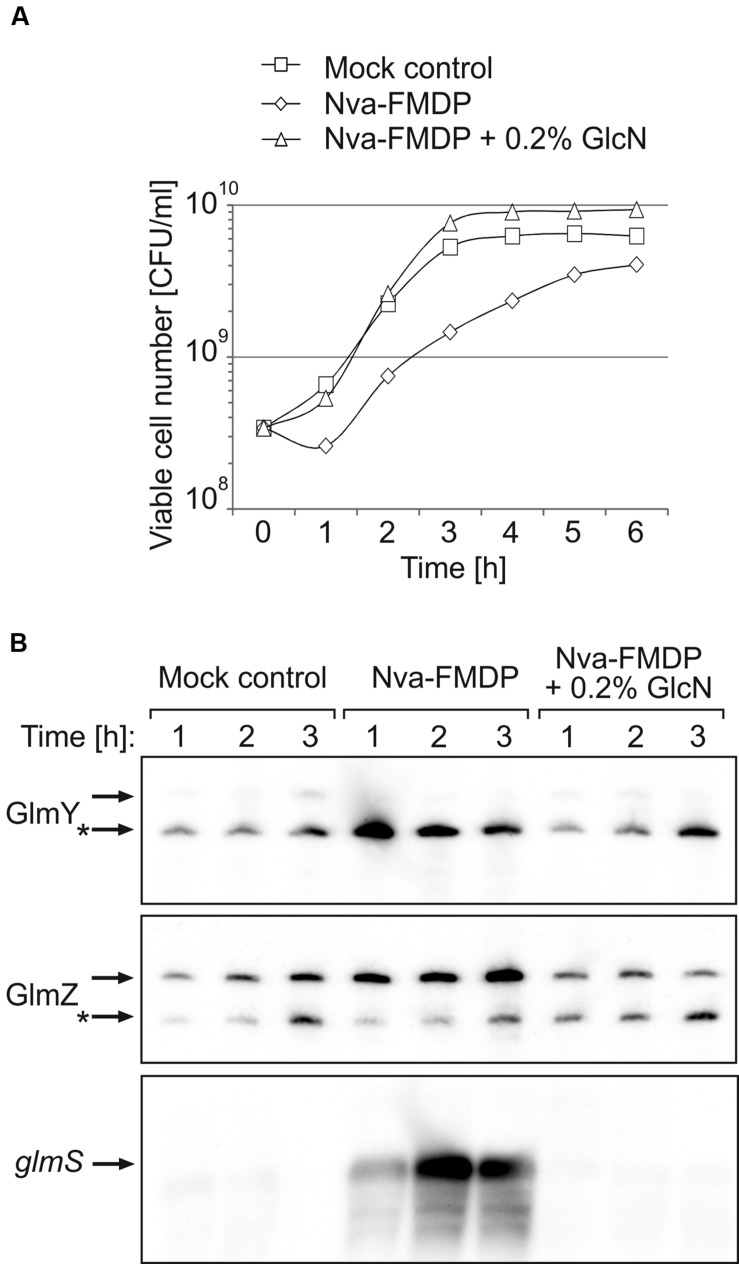
**Upregulation of GlmY, GlmZ, and *glmS* RNAs by Nva-FMDP also occurs in *Salmonella*. (A)** CFU numbers of cultures of *Salmonella* strain SL1344 grown in LB containing either 100 μg/ml Nva-FMDP (diamonds) or 100 μg/ml Nva-FMDP + 0.2% GlcN (triangles) or the equivalent volume of H_2_O (mock control; squares). The corresponding OD_600_ readings are provided in Supplementary Figure S5A. **(B)** Northern analysis of GlmY, GlmZ and *glmS* RNAs in the cultures analyzed in **(A)**. Total RNA was extracted from samples harvested in hourly intervals during the first 3 h after inoculation and subsequently probed for detection of GlmY (top panel), GlmZ (medium panel) and *glmS* RNAs (bottom panel). Loading controls are provided in Supplementary Figure S5B. The various RNA species are indicated by arrows. Processed variants are denoted by asterisks.

GlmY and GlmZ are conserved in *Enterobacteriaceae* and sequence analysis suggests that this even applies to GlmZ/*glmS* base-pairing (Fröhlich and Vogel, 2009; Göpel et al., 2011). This raises the possibility that GlmY and GlmZ may also bestow other *Enterobacteriaceae* with intrinsic resistance to GlmS inhibitors. To test this hypothesis, we investigated the effect of Nva-FMDP on the virulent *Salmonella enterica* serovar *Typhimurium* strain SL1344 and its isogenic *ΔglmY* and *ΔglmZ* mutants. First, we repeated the experiment shown in **Figure 2**, but used the *Salmonella* wild-type strain rather than *E. coli*. Indeed, Nva-FMDP elicited concomitant accumulation of GlmY and of the full-length variant of GlmZ (**Figure 6B**, top and medium panel). As expected, this resulted in upregulation of *glmS* mRNA (**Figure 6B**, bottom panel). The additional presence of GlcN suppressed accumulation of the various RNAs, recapitulating the observations in *E. coli* (compare **Figures 2B** and **6B**). Nva-FMDP had a significant negative effect on the viable cell number (**Figure 6A**), although this was not reflected by the OD_600_ readings (Supplementary Figure S5A), as observed for *E. coli* (**Figure 2A**; Supplementary Figure S1A).

Next, we studied susceptibility of *Salmonella* to Nva-FMDP and the roles of sRNAs GlmY and GlmZ in this process by carrying out disk diffusion assays and MIC determinations using wild-type strain SL1344 as well as the sRNA deletion strains (**Tables 2** and **3**; Supplementary Figures S4A,C). Globally, the results resembled those obtained for *E. coli*, but with some remarkable differences. First, Nva-FMDP had a stronger growth inhibitory effect on the *Salmonella* wild-type strain as compared to the *E. coli* wild-type. Zones of growth inhibition could be detected in all media (**Table 2**) and MIC values were generally lower for *Salmonella* decreasing in the order LB > Mueller Hinton > MacConkey lactose > M9 glucose (**Table 3**). Second, the absence of GlmY and GlmZ strongly increased susceptibility to Nva-FMDP confirming that these sRNAs provide intrinsic resistance even in *Salmonella*. Apparently, the sRNAs had a stronger impact on drug susceptibility in *Salmonella*: Depending on the medium, mutation of GlmY decreased the MIC 4- to 15-fold and mutation of GlmZ even 16- to 64-fold, whereas in *E. coli* a ~4-fold effect was seen (**Table 3**). In conclusion, GlmY and GlmZ are key players for the intrinsic resistance to antibiotics inhibiting GlmS, not only in *E. coli* but also in *Salmonella*.

### Combination with a Non-metabolizable GlcN6P Analog May Increase Efficacy of Nva-FMDP against the *E. coli* Wild-Type

**FIGURE 7:**
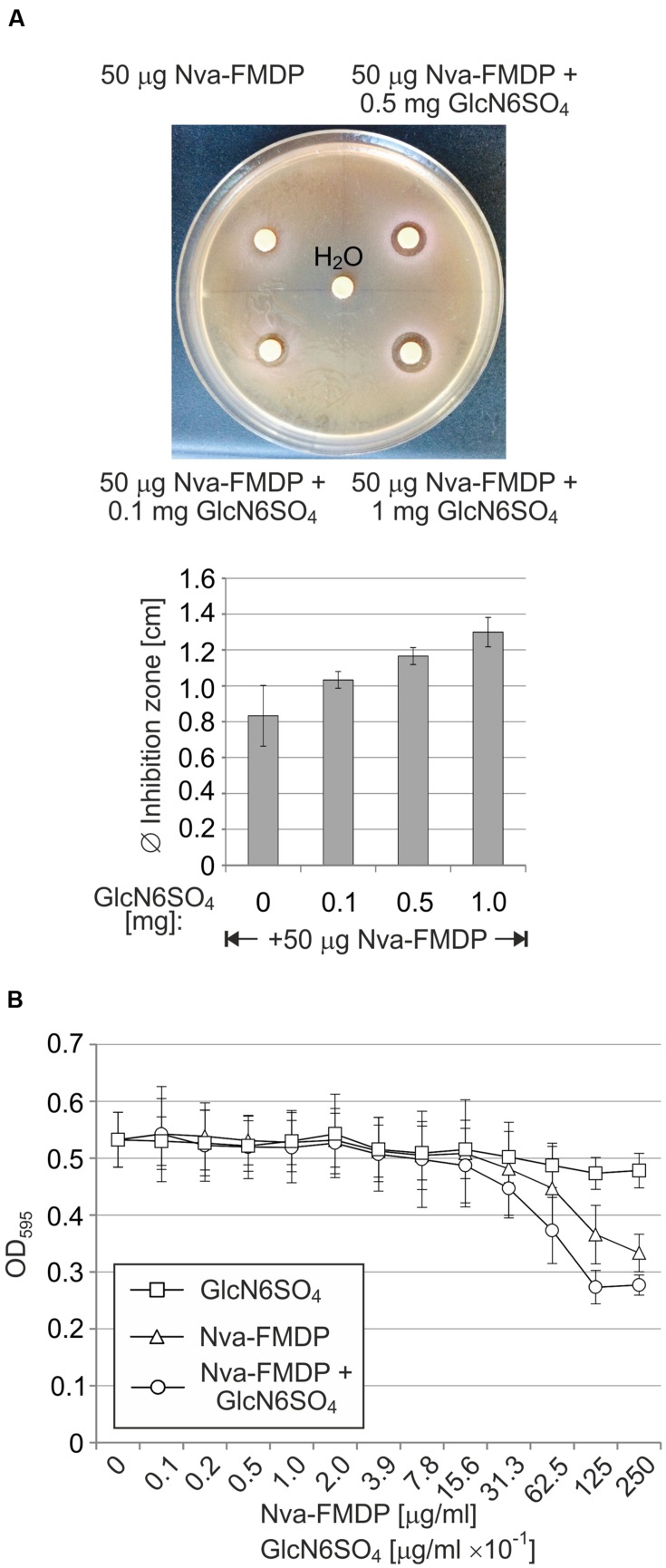
**Co-administration of GlcN6SO_4_ increases susceptibility of wild-type *E. coli* towards Nva-FMDP. (A)** Disk diffusion assay to address the combined effect of Nva-FMDP and GlcN6SO_4_ on *E. coli*. Filter disks containing 50 μg Nva-FMDP and various GlcN6SO_4_ amounts as indicated were applied to MacConkey lactose plates that were seeded with *E. coli wild-type* strain Z854. An exemplary experiment is shown at the top. A diagram depicting the results of three independent experiments is shown below. **(B)** Effect of GlcN6SO_4_ on susceptibility of *E. coli* to Nva-FMDP in LB liquid medium. Serial dilutions of Nva-FMDP or GlcN6SO_4_ or a mixture of both compounds were prepared in LB medium and subsequently inoculated with 5 × 10^5^ CFU/ml of *E. coli* strain Z854. Cell densities were recorded following 12 h incubation.

As GlmY and GlmZ are crucial for overcoming the deleterious effect of GlmS inhibitors, we reasoned whether suppression of the sRNAs could increase efficacy of these drugs against wild-type strains. Since the GlmY/GlmZ system apparently senses and responds to intracellular GlcN6P levels, we considered that a non-metabolizable analog of GlcN6P could potentially prevent the GlmY/GlmZ-mediated upregulation of GlmS, thereby increasing the efficacy of GlmS inhibition by Nva-FMDP. To provide a proof of concept, we used GlcN6SO_4_, which was shown to activate the *B. subtilis glmS* ribozyme to some extent *in vitro* indicating that it may act as GlcN6P analog in biological systems (Winkler et al., 2004). First, we tested GlcN6SO_4_ using disk diffusion assays. A small inhibition zone of ~8 mm diameter was observed when a filter disk loaded with 50 μg Nva-FMDP was applied onto a MacConkey lactose plate seeded with the *E. coli* wild-type strain Z854. Interestingly, the zone of growth inhibition gradually increased up to 13 mm upon co-administration of incremental amounts of GlcN6SO_4_ (**Figure 7A**). GlcN6SO_4_ showed no growth inhibitory properties on its own, indicating that it acts specifically by enhancing the antibacterial potential of Nva-FMDP (Supplementary Figure S6). Interestingly, this enhancing effect could not be observed for the *ΔglmY* and *ΔglmZ* mutant strains. In this case, constant inhibition zones were observed, regardless of the co-applied GlcN6SO_4_ potency, indicating that GlcN6SO_4_ requires the small RNAs to act (Supplementary Figure S7). To provide further evidence, we tested the combined effect of Nva-FMDP and GlcN6SO_4_ in liquid culture using the broth microdilution method. Serial dilutions of a solution containing 250 μg/ml Nva-FMDP and 2.5 mg/ml GlcN6SO_4_ were prepared in LB medium and subsequently inoculated with the *E. coli* wild-type strain Z854. As controls, serial dilutions containing only Nva-FMDP or GlcN6SO_4_ were tested in parallel. Indeed, following 12 h incubation, the cultures treated with the combination of both compounds showed a slightly stronger growth defect as compared to the cultures treated with Nva-FMDP alone (**Figure 7B**). Albeit weak, this difference was detectable in each individual experiment. The cultures that were solely supplemented with GlcN6SO_4_ exhibited no growth impairment confirming that this GlcN6P analog is nontoxic for the bacteria. Taken together, these results suggest that a GlcN6P analog such as GlcN6SO_4_ may have the potential to increase the antimicrobial activity of antibiotics targeting GlmS.

## Discussion

Peptide antibiotics targeting enzyme GlmS at the heart of bacterial cell envelope synthesis, are known for a long time. Structurally related oligopeptides such as Nva-FMDP have been developed, which lack the toxic effects exhibited by bacilysin on eukaryotic cells (Bontemps-Gracz et al., 1991). However, although readily taken up by peptide transporters, Gram-negative bacteria, such as *E. coli*, possess high intrinsic resistance towards these drugs, limiting their potential clinical use. Here, we reveal the mechanism underlying this intrinsic resistance. We show that both *E. coli* as well as *Salmonella* respond to these antibiotics by increased *glmS* expression, which compensates for inhibition of GlmS enzymatic activity (**Figures 2** and **6**). Cells respond in a dosage-dependent manner and adjust the level of *glmS* expression exactly to the level required to overcome growth inhibition by the GlmS inhibitor (**Figure 5**).

Responsible for this upregulation are two sRNAs which jointly regulate the *glmS* mRNA. Inhibition of GlmS by antibiotics leads to GlcN6P deprivation, which is sensed by sRNA GlmY triggering its accumulation (**Figures 2** and **4**). As expected, higher GlmY levels counteract degradation of sRNA GlmZ consequently activating *glmS* expression by base-pairing. We have recently shown that GlmY acts by sequestration of adapter protein RapZ, which otherwise binds GlmZ and recruits RNase E to inactivate the latter sRNA (Göpel et al., 2013, 2016). As these two sRNAs act hierarchically to control *glmS* expression, inactivation of either of them results in the inability to respond to GlcN6P deprivation and consequently increases susceptibility to GlmS inhibitors significantly, i.e., up to eightfold for *E. coli* and up to 64-fold for *Salmonella* depending on the growth medium (**Tables 2** and **3**). The basis for the different impact of the sRNAs on drug susceptibility in these two species is unknown, but could be caused by differences in basal *glmS* expression levels remaining in the absence of GlmY or GlmZ. The reason for the strong variation of efficacy of Nva-FMDP by the growth medium also remains unclear. A reasonable explanation might be provided by competition of Nva-FMDP with externally available peptides for uptake (Marshall et al., 2003) and/or by the known repression of the dipeptide transporter component DppA by amino acids (Olson et al., 1991). Regardless of the growth medium, GlmY/GlmZ endow both species with intrinsic resistance against GlmS inhibitors. Thus, this mechanism might be conserved in all species possessing GlmY/GlmZ, i.e., in *Enterobacteriaceae*.

Our data indicate that suppression of the GlmY/GlmZ circuit would substantially increase susceptibility of wild-type bacteria to GlmS inhibitors. As the GlmY/GlmZ cascade is sensing and responding to GlcN6P, a non-metabolizable analog should accomplish this task. Initial experiments using GlcN6SO_4_ support this possibility as this compound increases efficacy of Nva-FMDP in a GlmY/GlmZ-dependent manner, albeit weakly (**Figure 7**; Supplementary Figure S7). The reason for the weak activity of GlcN6SO_4_ is unclear, but hydrolysis by so far unknown sulfatases or inefficient uptake is a likely explanation. Thus, a future task will be the identification of a stable GlcN6P analog that suppresses the GlmY/GlmZ system efficiently. Testing the numerous artificial GlcN6P mimics that have been synthesized to serve as artificial actuators of the Gram-positive *glmS* ribozyme (Fei et al., 2014), might provide a good starting point. Finally, it might also be possible to design this analog on a rational basis, once the GlcN6P sensor and the way how it interacts with the amino sugar has been identified. As it sits at the top of the regulatory cascade, sRNA GlmY is the most likely candidate, a possibility that is currently addressed in our laboratory.

The contribution of RNA regulatory elements to antibiotic resistance and their potential value as drug targets is appreciated only recently. Traditionally, screens for novel antimicrobial targets or factors mediating drug resistance have focused on proteins (Martinez and Rojo, 2011; Cox and Wright, 2013). A first systematic screen investigating the roles of various Hfq-dependent sRNAs for susceptibility to different classes of antibiotics has been reported recently (Kim et al., 2015). Several sRNAs were shown to alter antibiotic susceptibility in *E. coli* and *Salmonella* when deleted and/or overproduced. The underlying mechanisms remain largely unknown, but in two cases regulation of eﬄux pump components by sRNAs might be involved (Nishino et al., 2011; Kim et al., 2015; Parker and Gottesman, 2016). In sum, there is increasing evidence that sRNAs have crucial roles in genetic networks that provide intrinsic resistance to antibiotics, either by acting via more unspecific mechanisms (e.g., drug eﬄux pumps) and involving multiple targets (Schnorpfeil et al., 2013; Kim et al., 2015) or by impacting specifically on a single gene whose product is selectively targeted by a cognate antibiotic (this study). It remains to be investigated, whether sRNAs and other regulatory RNA elements indeed represent druggable targets proving useful for antimicrobial chemotherapy. In this regard, a recent study is encouraging that identified a compound, which binds to an RNA riboswitch and thereby suppresses synthesis of the essential vitamin riboflavin (Howe et al., 2015). Future work must determine whether the same holds true for the GlmY/GlmZ system, i.e., whether a compound could be identified that interferes with the sRNAs and inhibits their response to GlcN6P deprivation, thereby increasing susceptibility to GlmS inhibitors in combined therapy.

## Author Contributions

BG designed the study. BG and MK conceived the experiments. MK performed the experiments with assistance of YG. All authors analyzed the data and discussed the results. SM contributed material. BG wrote the paper. All authors read, commented on and approved the final manuscript.

## Conflict of Interest Statement

The authors declare that the research was conducted in the absence of any commercial or financial relationships that could be construed as a potential conflict of interest.
