# Supplementary material for: Two Small RNAs Conserved in Enterobacteriaceae Provide Intrinsic Resistance to Antibiotics Targeting the Cell Wall Biosynthesis Enzyme Glucosamine-6-Phosphate Synthase
Source: Front Microbiol. 2016 Jun 15;7:908. doi: 10.3389/fmicb.2016.00908 (PMC4908143; doi:10.3389/fmicb.2016.00908)
Supplement: Supplementary file 1 [file Data_Sheet_1.DOCX]

Supplementary Material

**Two Small RNAs Conserved in *Enterobacteriaceae* Provide Intrinsic Resistance to Antibiotics Targeting the Cell Wall Biosynthesis Enzyme Glucosamine-6-phosphate Synthase**

Muna Ayesha Khan^1^, Yvonne Göpel^1^, Slawomir Milewski^2^, and Boris Görke^1*^

^1^Department of Microbiology, Immunobiology and Genetics, Max F. Perutz Laboratories (MFPL), University of Vienna, Vienna Biocenter (VBC), Vienna, Austria, ^2^Department of Pharmaceutical Technology and Biochemistry, Faculty of Chemistry, Gdańsk University of Technology, Gdańsk, Poland.

Running title: sRNAs GlmYZ as targets for antimicrobial chemotherapy

^*^Correspondence:

Boris Görke

[boris.goerke@univie.ac.at](https://webmail.univie.ac.at/src/compose.php?send_to=boris.goerke%40univie.ac.at).

## Supplementary Figures


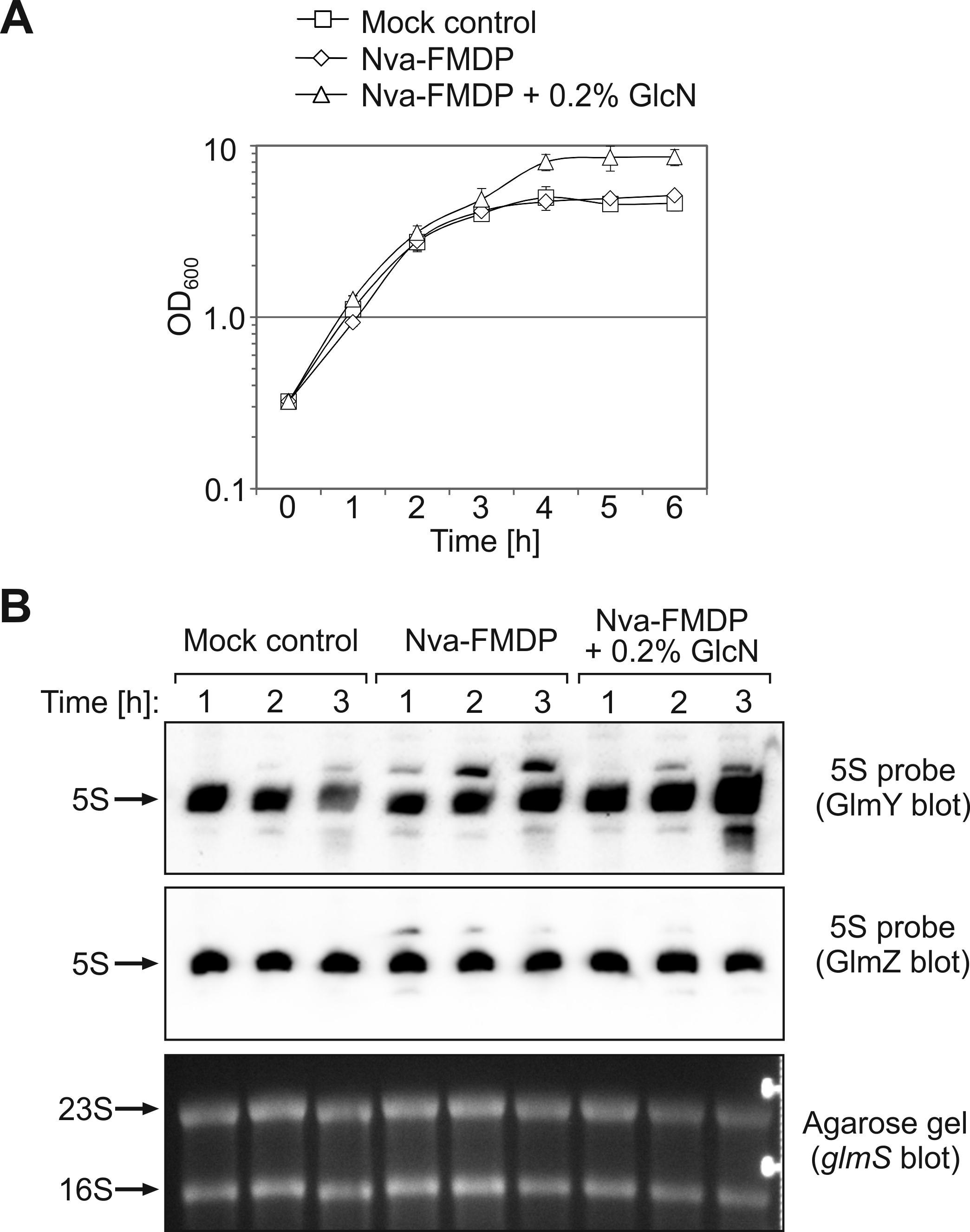


**Supplementary Figure 1.** **Controls for the experiment shown in Figure 2.** (A) OD_600_ recordings of the cultures analysed in Figure 2 A. (B) Loading controls for Northern blots presented in Figure 2 B. The blots addressing GlmY and GlmZ sRNAs were re-probed using a probe specific for 5S rRNA encoded by gene *rrfD*. In addition, the ethidium bromide-stained agarose gel corresponding to the *glmS* blot in Figure 2 B is shown.


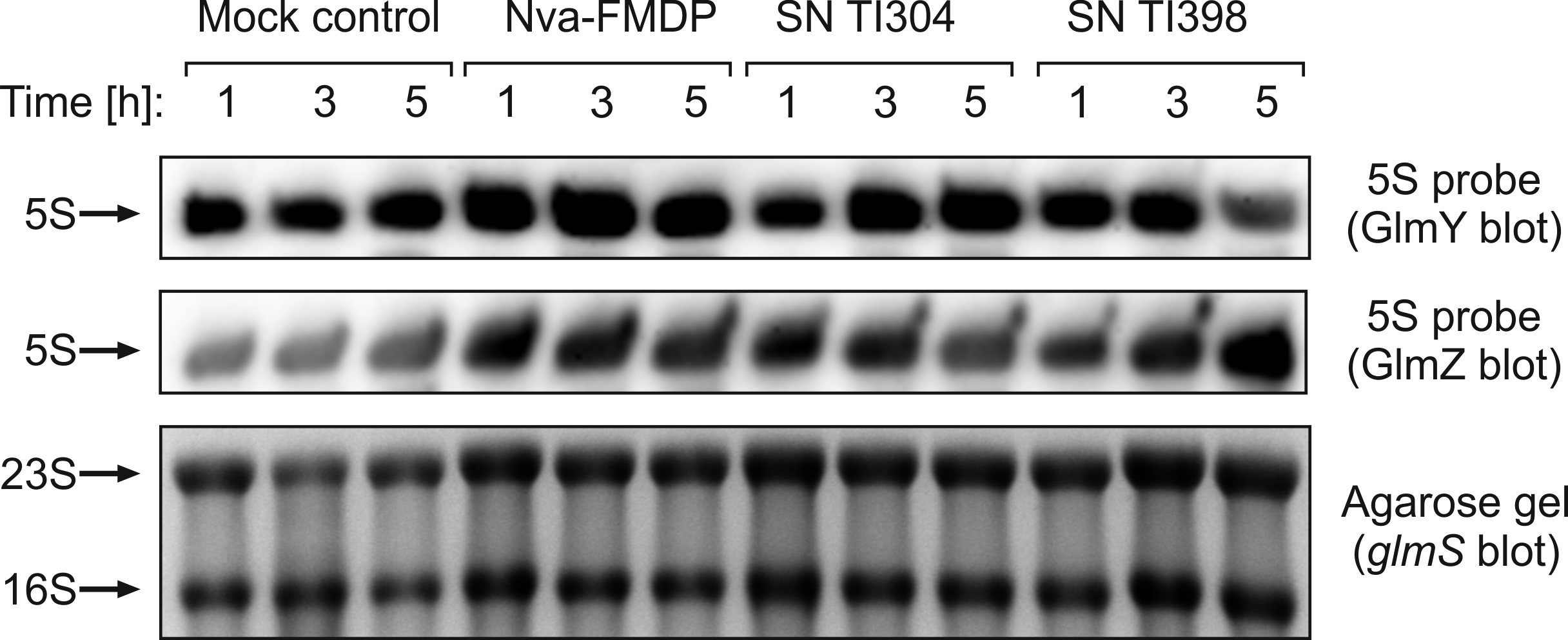


**Supplementary Figure 2.** **Loading controls for Northern blots presented in Figure 3 B.** The blots addressing GlmY and GlmZ sRNAs were re-probed using a probe specific for 5S rRNA encoded by gene *rrfD*. In addition, the ethidium bromide-stained agarose gel corresponding to the *glmS* blot in Figure 3 B is shown.

**
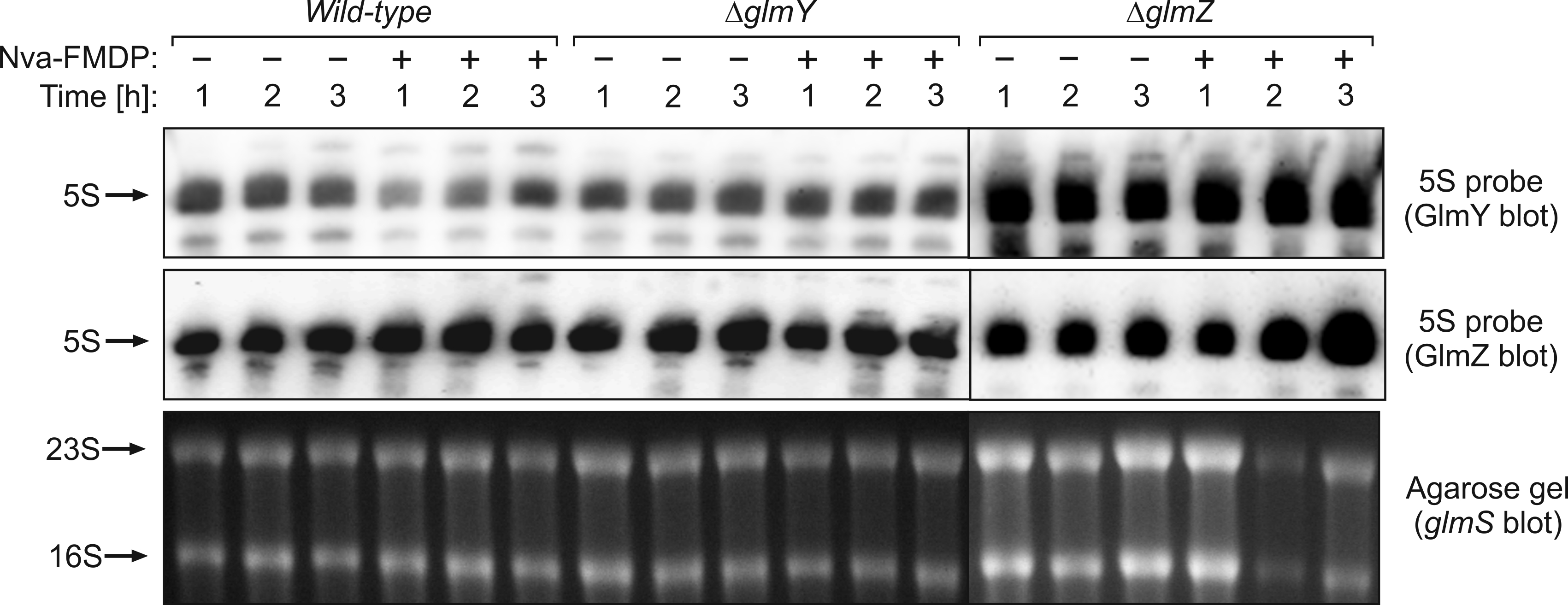
**

**Supplementary Figure 3.** **Loading controls for Northern blots presented in Figure 4 C.** The blots addressing GlmY and GlmZ sRNAs were re-probed using a probe specific for 5S rRNA encoded by gene *rrfD*. In addition, the ethidium bromide-stained agarose gel corresponding to the *glmS* blot in Figure 4 C is shown.


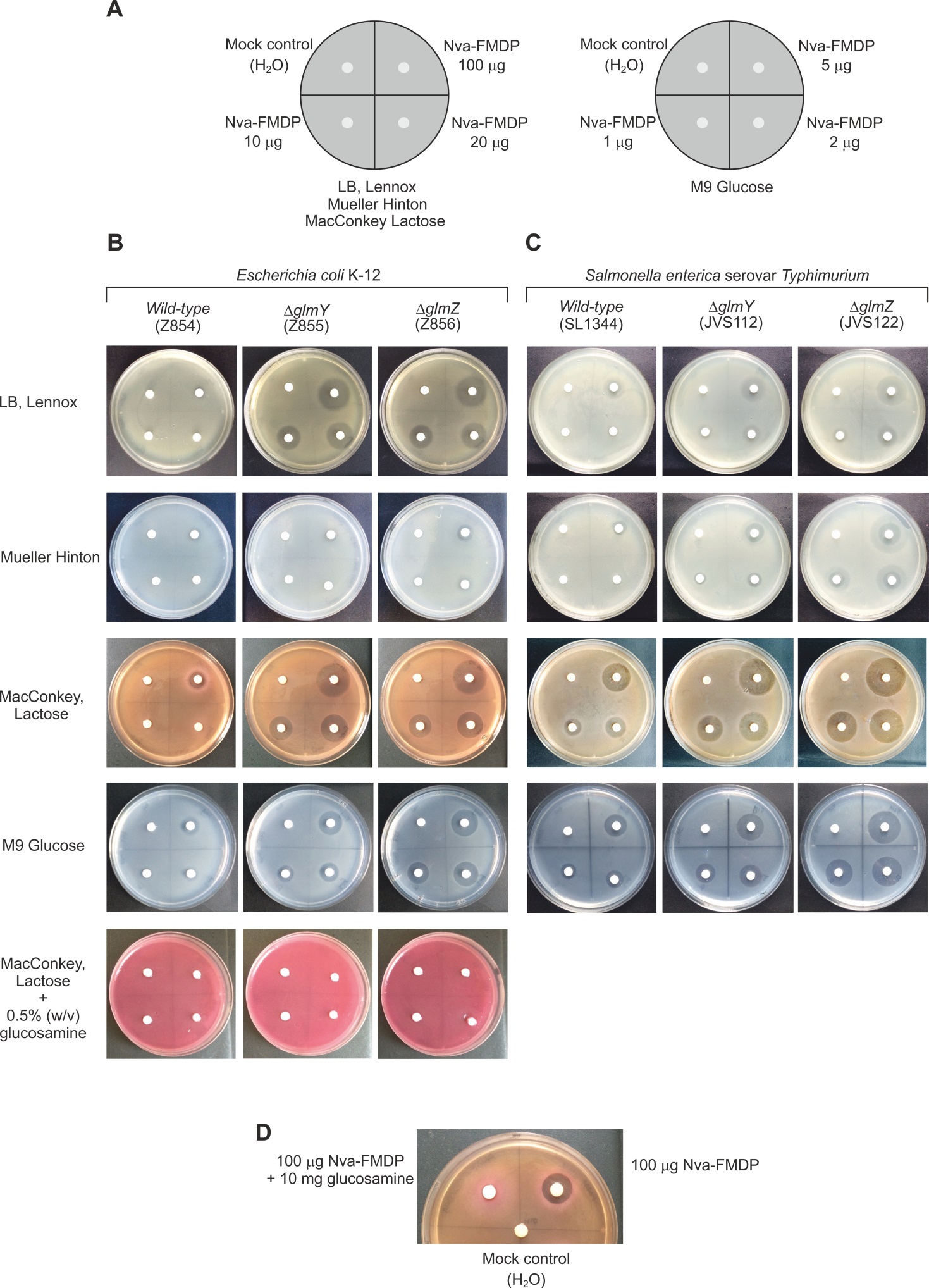


**Supplementary Figure 4.** **Disk diffusion assays for testing efficacy of Nva-FMDP against *E. coli* and *Salmonella* strains in various media** (Representative set of assays that contributed as source data to the values in Table 2). (A) Schematic representation depicting the arrangement of the differently loaded filter disks on the agarose plates shown in (B) and (C). Filter disks containing Nva-FMDP amounts ranging from 10 – 100 μg were used to test activity in LB, Mueller Hinton and MacConkey lactose media. Nva-FMDP amounts ranging from 1 – 5 μg were applied to M9 glucose minimal plates. (B) Disk diffusion assays were carried out using *E. coli* strains Z854 (*wild-type*), Z855 (*ΔglmZ*) and Z856 (*ΔglmY*). In the last row, MacConkey lactose plates supplemented with 0.5 % GlcN were tested (C) Disk diffusion assays addressing susceptibility of *Salmonella* strains SL1344 (*wild-type*), JVS112 (*ΔglmY*) and JVS122 (*ΔglmZ*). (D) Co-administration of GlcN suppresses growth inhibition of *E. coli* strain Z854 (wild-type) by Nva-FMDP. 10 mg GlcN and 100 μg Nva-FMDP were co-applied directly onto the filter disk (top, left). A filter disk containing only 100 μg Nva-FMDP served as control (top, right).


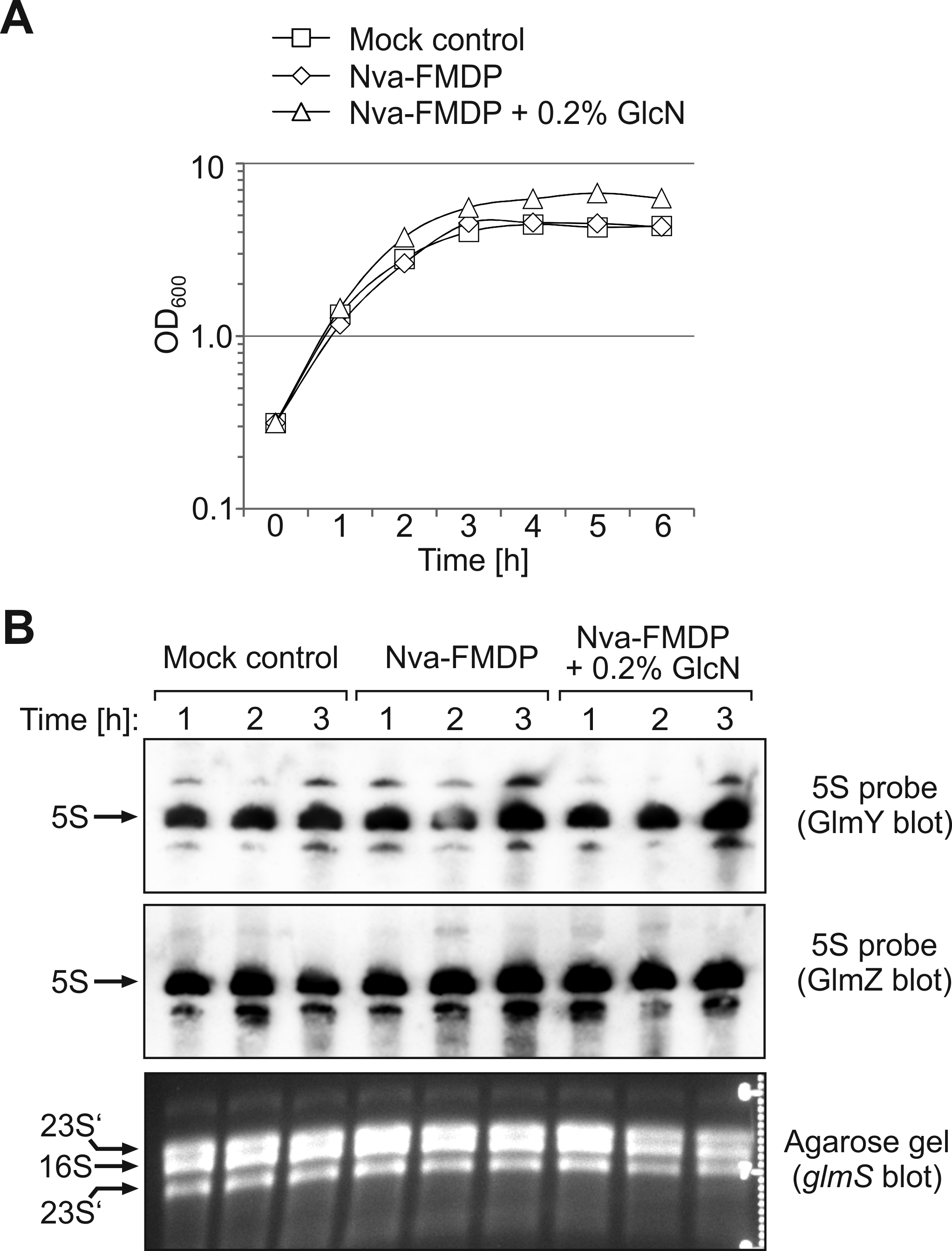


**Supplementary Figure 5.** **Controls for the experiment shown in Figure 6.** (A) OD_600_ recordings of the cultures analysed in Figure 6 A. (B) Loading controls for Northern blots presented in Figure 6 B. The blots addressing GlmY and GlmZ sRNAs were re-probed using a probe specific for 5S rRNA encoded by gene *rrfD*. In addition, the ethidium bromide-stained agarose gel corresponding to the *glmS* blot in Figure 6 B is shown. Note that 23S rRNA is cleaved in *Salmonella* resulting in 18S and 14S fragments (D. Hsu, Y.C. Zee, J. Ingraham and L.M. Shih, J Gen Microbiol **138:**199-203, 1992, doi: [10.1099/00221287-138-1-199](http://dx.doi.org/10.1099/00221287-138-1-199)).


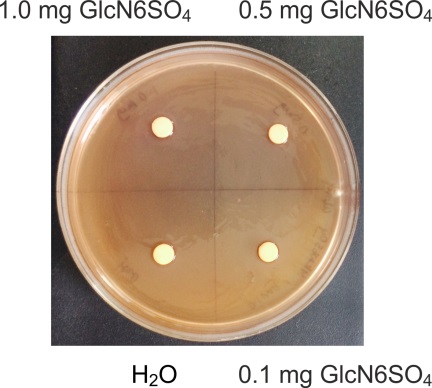


**Supplementary Figure 6.** **GlcN6SO_4_ lacks growth inhibition properties towards *E. coli* in disk diffusion assays.** Filter disks containing the indicated GlcN6SO_4_ amounts were applied to a MacConkey lactose plate that was seeded with the *E. coli wild-type* strain Z854.


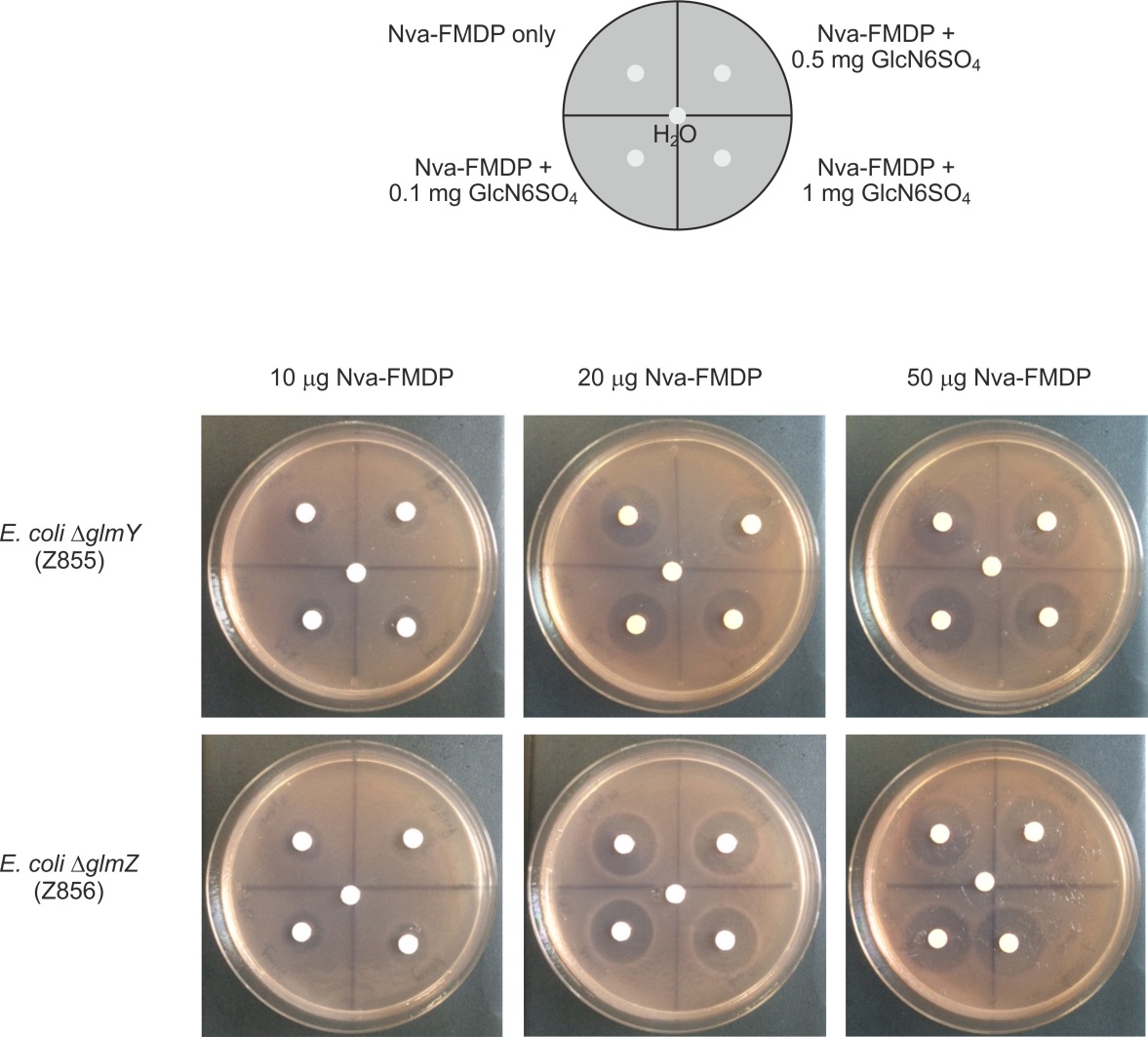


**Supplementary Figure 7.** **Co-administration of GlcN6SO_4_ is without effect on susceptibility of *E. coli* *ΔglmY* and *ΔglmZ* mutants to Nva-FMDP.** Disk diffusion assays addressing the combined effect of Nva-FMDP and GlcN6SO_4_ on *E. coli ΔglmY and ΔglmZ* mutants. Filter disks containing 10 μg, 20 μg or 50 μg Nva-FMDP and various amounts of GlcN6SO_4_ as indicated in the schematic representation at the top were applied onto MacConkey lactose plates that were either seeded with *E. coli* strain Z855 (*ΔglmY*; top row) or strain Z856 (*ΔglmZ*; bottom row).
